# Homocysteine, Nutrition, and Gut Microbiota: A Comprehensive Review of Current Evidence and Insights

**DOI:** 10.3390/nu17081325

**Published:** 2025-04-11

**Authors:** Deborah Agostini, Alessia Bartolacci, Rossella Rotondo, Maria Francesca De Pandis, Michela Battistelli, Matteo Micucci, Lucia Potenza, Emanuela Polidori, Fabio Ferrini, Davide Sisti, Francesco Pegreffi, Valerio Pazienza, Edy Virgili, Vilberto Stocchi, Sabrina Donati Zeppa

**Affiliations:** 1 Department of Biomolecular Sciences, University of Urbino Carlo Bo, 61029 Urbino, Italy; deborah.agostini@uniurb.it (D.A.); michela.battistelli@uniurb.it (M.B.); matteo.micucci@uniurb.it (M.M.); lucia.potenza@uniurb.it (L.P.); emanuela.polidori@uniurb.it (E.P.); fabio.ferrini@uniurb.it (F.F.); davide.sisti@uniurb.it (D.S.); sabrina.zeppa@uniurb.it (S.D.Z.); 2 Department of Human Science and Promotion of Quality of Life, San Raffaele Rome Open University, 00166 Rome, Italy; francesca.depandis@uniroma5.it (M.F.D.P.); vilberto.stocchi@uniroma5.it (V.S.); 3 San Raffaele Cassino, 03043 Cassino, Italy; 4 Department of Medicine and Surgery, Kore University of Enna, 94100 Enna, Italy; francesco.pegreffi@unikore.it; 5 Division of Gastroenterology, “Casa Sollievo della Sofferenza” Hospital, 71013 San Giovanni Rotondo, Italy; v.pazienza@operapadrepio.it; 6 School of Biosciences and Veterinary Medicine, University of Camerino, 62031 Camerino, Italy; info@edyvirgili.it

**Keywords:** homocysteine, gut microbiota, diet, supplements, folic acid, vitamin B12

## Abstract

Homocysteine, a sulfur-containing amino acid, is an intermediate product during the metabolism of methionine, a vital amino acid. An elevated concentration of homocysteine in the plasma, named hyperhomocysteinemia, has been significantly related to the onset of several diseases, including diabetes, multiple sclerosis, osteoporosis, cancer, and neurodegenerative disorders such as dementia, Alzheimer’s and Parkinson’s diseases. An interaction between metabolic pathways of homocysteine and gut microbiota has been reported, and specific microbial signatures have been found in individuals experiencing hyperhomocysteinemia. Furthermore, some evidence suggests that gut microbial modulation may exert an influence on homocysteine levels and related disease progression. Conventional approaches for managing hyperhomocysteinemia typically involve dietary interventions alongside the administration of supplements such as B vitamins and betaine. The present review aims to synthesize recent advancements in understanding interventions targeted at mitigating hyperhomocysteinemia, with a particular emphasis on the role of gut microbiota in these strategies. The emerging therapeutic potential of gut microbiota has been reported for several diseases. Indeed, a better understanding of the complex interaction between microbial species and homocysteine metabolism may help in finding novel therapeutic strategies to counteract hyperhomocysteinemia.

## 1. Introduction

### Homocysteine and Gut Microbiota

Homocysteine (Hcy), a sulfur-containing amino acid, is present in human plasma at low concentrations. Hcy is involved in two key metabolic pathways: remethylation to L-Methionine (Met) and transsulfuration to L-cystathionine. Remethylation requires folate and vitamin B12 (or betaine as an alternative), while transsulfuration requires pyridoxal-5′-phosphate (PLP). These pathways are regulated by S-adenosylmethionine (SAM), which inhibits the methylenetetrahydrofolate reductase (MTHFR) reaction and activates cystathionine β-synthase (CBS) [1].

Increased levels of plasma Hcy is a status referred to as hyperhomocysteinemia (HHcy). Abnormally high levels of Hcy have important implications for human health and disease. Clinical studies indicate that HHcy is an independent risk factor for cardiovascular diseases (CVDs) and can promote glucose intolerance, insulin resistance, and hepatic steatosis and is closely linked with an enhanced vulnerability to CVD, ischemic and pediatric hemorrhagic stroke [2], and recently, adult-onset Alzheimer’s and other neurological impairments [3,4,5,6]. Factors affecting Hcy levels include genetic deficiencies, high Met intake, lack of vitamins B6/B12 or folic acid (FA), and certain drugs [7]. Genetic anomalies in Met biological transformations, notably those involving methionine synthase (MS) and N5, N10-methylenetetrahydrofolate, have been linked to neurological imbalance, intellectual impairments, and pregnancy problems [8]. Furthermore, current investigations have observed connections between homocysteine (Hcy), folate, and DNA methylation in the scenario of gut and inflammation-related illnesses [9,10]. The postulated mechanism connecting Hcy to inflammation involves the activation of leukocyte adhesion molecules and the release of proinflammatory mediators secreted by vascular endothelium [11].

Additionally, HHcy has been demonstrated to suppress cell proliferation and induce inflammatory reactions in endothelial cells (EC), thereby compromising endothelial function, which is a critical indicator of vascular damage [12].

The liver is the primary site of Met and Hcy metabolism, but recent studies suggest the gastrointestinal tissue (GIT) also plays a role. The gastrointestinal tract contains a complex community of microbial cells, referred to as the gut microbiota, which has coevolved with the human organism and has been recognized as a crucial determinant of host health [13].

The gut microbiota influences host health, can affect amino acid metabolism, and participates in various physiological processes, such as educating the host’s immune system, synthesizing vitamins, regulating nutrient absorption and metabolism, providing protection against pathogens, and regulating intestinal endocrine functions [14].

Its composition is inherently variable and influenced by a range of environmental and behavioral variables, including age, dietary habits, supplementation, and physical activity [15,16,17,18,19]. These factors collectively impact the metabolic pathways of the host. Emerging research has highlighted the potential interactions between gut microbiota and Hcy metabolism, suggesting that alterations in microbial communities may contribute to elevated Hcy levels and associated metabolic disorders. Understanding the interplay between Hcy and the gut microbiota is essential for developing novel therapeutic strategies to mitigate the health risks associated with HHcy.

In this review, the bidirectional influence of Hcy level and gut microbiota composition, with particular attention on the effects of diet and supplements on normal levels of Hcy maintenance, will be discussed.

## 2. Homocysteine Metabolism

**Figure 1:**
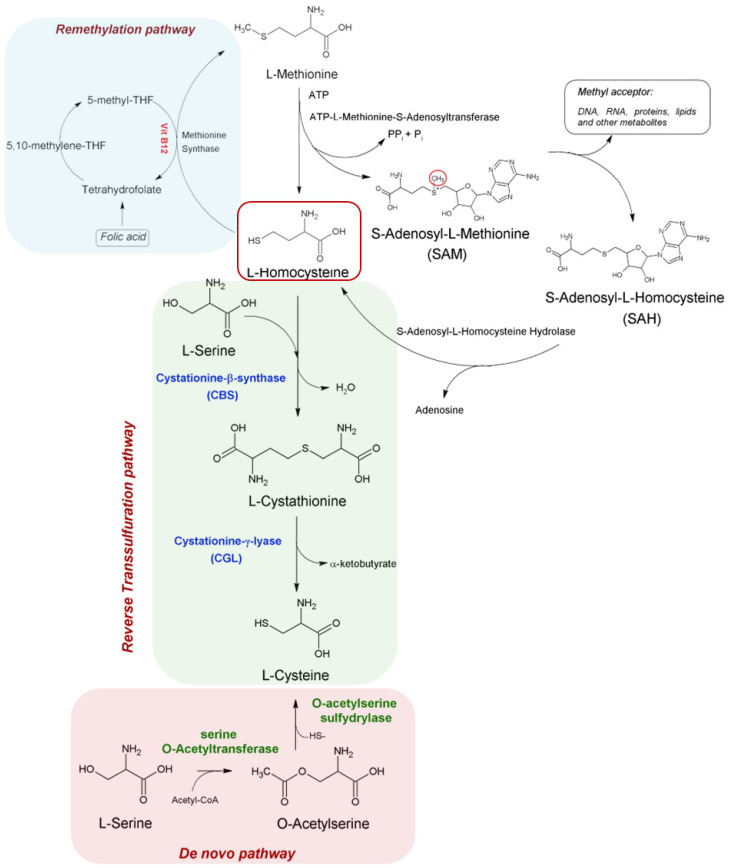
Schematic diagram of Hcy biosynthesis, including the metabolic processes of reverse transsulfuration pathway and de novo pathway and re-methylation associated with the folate cycle. L-Methionine is activated by ATP in a reaction catalyzed by methionine S-adenosyl-transferase with the concomitant hydrolysis of ATP and formation of S-Adenosyl-L-Methionine (SAM). The latter is demethylated into S-adenosyl-L-homocysteine (SAH) coupled with methylation of an acceptor -R into -RCH_3_. SAH is hydrolyzed into L-homocysteine (Hcy) and adenosine by S-adenosyl-L-homocysteine hydrolase. In the reverse transsulfuration pathway, the condensation of L-serine (L-Ser) with Hcy forms L-cystathionine through the PLP-dependent cystathionine β-synthase (CBS) activity, followed by the conversion of L-cystathionine into cysteine (Cys) and α-ketobutyrate catalyzed by PLP– dependent cystathionine-γ-lyase (CGL). Demethylation of N5-methyl-tetrahydrofolate (5-methyl-THF) into THF and concomitant Hcy remethylation into L-Methionine by methionine synthase dependent on vitamin B12. The reduction of 5,10-methylene-THF into 5-methyl-THF is catalyzed by N5,10-methylenetetrahydrofolate reductase (MTHFR). In bacteria and plants, the de novo pathway forms L-cysteine from L-serine through the intermediate O-acetylserine by two tandem reactions mediated by serine O-acetyl transferase (SAT) and O-acetylserine sulfhydrylase (OASS).

Homocysteine is not supplied by the diet; however, it is an essential intermediate of Met metabolism in mammals. Each metabolite, Met or Hcy, serves as a precursor of the other. Their synthesis and detoxification are closely related since the synthesis of one represents the detoxification mechanism of the other. The Met cycle is a ubiquitous process: in some tissues, Hcy is diverted from the cycle to increase the biosynthesis of cysteine and its derivatives by the transsulfuration pathway (Figure 1). Methionine metabolism, therefore, depends on Hcy distribution between the two competing pathways [20].

### 2.1. Biosynthesis and Metabolism of Homocysteine

Homocysteine biosynthesis is a multistep process, starting from demethylation of Met [1]. In the first step, ATP-L-Methionine S-Adenosyltranferase (MAT) catalases the transfer of the adenosyl group of ATP to methionine, leading to the formation of S-Adenosyl-L-Methionine (AdoMet or SAM). This latter metabolite–often referred to as the universal methyl donor–transfers its methyl group to a wide range of acceptor molecules, including DNA, RNA, proteins, lipids and other metabolites [21]. The reaction product, S-Adenosyl homocysteine (AdoHCys or SAH), is subsequently hydrolyzed to Hcy and adenosine by S-adenosyl homocysteine hydrolase (AHCY). Although the AdoHCys synthesis is thermodynamically favored, the fast demand and consumption of Hcy and adenosine shift the equilibrium of the reaction towards hydrolysis [22]. Hcy is a key intermediate of two central metabolic pathways: recycling into L-Methionine via tetrahydrofolate (THF) or conversion to L-cysteine [22].

### 2.2. Remethylation Pathway

Remethylation of Hcy to L-Methionine is catalyzed by the cobalamin-dependent methionine synthase (MS) by using 5-N-methyl tetrahydrofolate (5-methyl-THF) as a methyl donor [22]. Despite 5- N-methyl-THF being the major source of methyl groups for the remethylation of Hcy, in certain conditions, betaine can also act as methyl group donors via betaine-homocysteine methyltransferase (BHMT) [22]. However, considering the gene expression of BHMT, the liver and kidney are the primary sites for the betaine pathway, where it represents an intermediate for choline oxidation [22].

### 2.3. Reverse Transsulfuration and De Novo Pathways

The interconversion of Hcy to L-cysteine is mediated by the transsulfuration pathway, a metabolic route involving L-cystathionine as an intermediate. Two transsulfuration pathways are known: the de novo pathway and the reverse pathway [23].

The transsulfuration pathway in mammals operates in the opposite direction from microbes, therefore it is generally referred to as the reverse transsulfuration pathway.

The conversion of Hcy to L-cysteine is a well-known two-step process, proceeding from the condensation of Hcy with L-serine to form cystathionine in a reaction catalyzed by cystathionine β-synthase (CBS). In the second step, the cystathionine is converted to cysteine and α-ketobutyrate by cystathionine γ-lyase (CGL) [24]. Both enzymes are PLP-dependent enzymes, rendering the pathway sensitive to vitamin B6 status. CBS and CGL are highly expressed in the liver, pancreas and kidney [22,25].

In bacteria and plants, the de novo pathway forms L-cysteine from L-serine [26,27,28].

Evidence suggests the presence of reverse transsulfuration pathways also in certain bacteria, where the de-novo and reverse transsulfuration pathways seem interconnected [29,30,31].

## 3. The Relationship Between Diet and Homocysteine

Homocysteine is not naturally present in food, but it is an essential intermediate in the normal mammalian metabolism of methionine, which is closely related to the availability of B vitamins, particularly folate, vitamin B12, and vitamin B6.

Diet plays a crucial role in modulating Hcy levels and the composition of the gut microbiota. In healthy individuals, the physiological levels of Hcy are primarily influenced by the intake of methionine, folate, and vitamin B12 in their diet. A diet lacking these vitamins can lead to reduced enzyme activities, inhibiting the breakdown of Hcy and thus increasing the intracellular Hcy concentration. Conversely, supplementation with folate, vitamin B12, and vitamin B6 can reduce Hcy levels and improve cardiovascular health [32]. Additionally, lifestyle factors such as excessive consumption of coffee or alcohol, smoking, and lack of physical activity can affect Hcy metabolism and plasma levels. Consequently, an imbalance in Hcy levels can lead to HHcy, a condition characterized by elevated plasma Hcy levels, with normal values typically ranging from 5 to 15 µmol/L. Elevated Hcy levels are categorized as mild (15 to 30 µmol/L), moderate (30 to 100 µmol/L), and severe (greater than 100 µmol/L) [33]. Severe HHcy occurs in classical homocystinuria, a genetic disorder caused by CBS deficiency, while mild HHcy has been associated with the thermolabile variant of MTHFR, deficiencies in B complex vitamins, certain medications, aging, lifestyle factors, and air pollution [34,35,36].

HHcy is associated with an increased risk of CVDs, with toxic effects demonstrated in various in vivo and in vitro models, particularly concerning neural cells [37].

Foscolou et al. (2019) investigated the relationship between Hcy level and acute coronary syndrome (ACS), as well as assessing the potential moderating role of the Mediterranean Diet (MD). The study encompassed 1491 patients experiencing their first ACS event and 3037 adults without any CVD. The findings revealed an inverse association between adherence to the MD and Hcy levels, underscoring the disease-preventing effect of the MD on CVD [38].

It is known that insufficient intake of FA, vitamin B6, and vitamin B12 leads to higher Hcy levels but the relationship between Hcy and other dietary factors is not clear. The association between Hcy and nutrient intake was investigated by Tajima et al. conducting a dietary survey among 227 young women over seven consecutive days, complemented by digital imaging [39]. The results of this study revealed a significant negative association between serum Hcy concentrations and intake of soluble, insoluble, and total fiber. Additionally, higher consumption of fruits and mushrooms was observed to reduce serum Hcy levels among the various food groups, suggesting that dietary fiber from these foods may play a role. Conversely, no significant association was found between Hcy level and intake of cereals and vegetables. These findings indicate that differences in fiber quality, possibly due to antioxidant components such as polyphenols from fruits, along with antioxidant and anti-inflammatory compounds derived from mushrooms, may contribute to the observed effects.

Dietary monitoring and Hcy levels in 286 women aged between 23 and 46 years with fertility disorders revealed that the mean percentage of monounsaturated fatty acids (MUFAs) and polyunsaturated fatty acids (PUFAs) in total energy intake was significantly lower in diets of women with Hcy level > 15 μmol/L compared to those with Hcy < 10 μmol/L.

The observed correlations suggest that a higher intake of PUFAs, such as α-linoleic acid, appears to be a crucial factor in preventing HHcy [40].

The impact of obesity on hepatic Hcy metabolism in male C57BL/6 mice fed a high-fat diet for 12 weeks, has been investigated by Yun et al. [41]. The study found that male C57BL/6 mice fed a high-fat diet showed an increased plasma Hcy level but a decreased hepatic Hcy level. Obese mice have reduced AHCY levels, leading to a decrease in transmethylation potential. Taurine synthesis is activated through cysteine dioxygenase up-regulation. Elevated plasma Hcy levels in obesity-related fatty liver disease may be due to increased hepatic Hcy efflux and altered sulfur amino acid metabolism.

Transmethylation processes and antioxidant activity are interconnected through transsulfuration, a pathway in which homocysteine (Hcy) is transformed into cysteine and subsequently into reduced glutathione (GSH). This metabolic pathway is susceptible to modulation by dietary factors, such as reduced protein consumption, which can influence the efficiency of these reactions. The effect of a low-protein diet on Hcy metabolism was evaluated by monitoring levels of the amino acids involved in these pathways, and relating these levels to GSH levels and lipid peroxidation in rats. Hcy levels were reduced under a low-protein diet, resulting in modulated methyl balance and reduced GSH formation leading to increased susceptibility of hepatic cells to oxidative events [42].

The relation between dietary protein intake and plasma total HCy, and cysteine concentrations in 1015 coronary angiographic Chinese patients has been investigated out by Xiao et al. [43].

The results of this study indicated that high animal-protein and total-protein intakes were positively associated with plasma tHcy and tCys concentrations, whereas plant-protein intake was a negative determinant of plasma tHcy concentrations.

The results of this study revealed a positive correlation between an enhanced intake of animal-related and total protein and increased plasma concentrations of tHcy and tCys. Conversely, intake of plant-based protein was identified as a negative predictor of plasma tHcy levels. Methionine plays a key role in protein metabolism, highlighting its importance for growth and maintaining lean body mass. This makes Met an attractive option for supplementation. However, since Met is also a precursor to Hcy, a lack of B vitamins or an excessive intake of Met could lead to HHcy, which is a risk factor for CVD.

Supplementation with dietary methionine led to elevated plasma homocysteine (Hcy) levels, a decreased ratio of reduced glutathione (GSH) to oxidized glutathione (GSSG), reduced GSH level, increased proinflammatory cytokine formation, and suppressed expression of apolipoprotein B (apoB). These effects contributed to lipid accumulation within cardiac tissue in mice. In rats, mild to moderate hyperhomocysteinemia (HHcy) was associated with increased systolic blood pressure following sympathomimetic stimulation. Interestingly, despite exhibiting vascular endothelial dysfunction, CBS knockout mice with severe HHcy showed no prothrombotic risk despite developing vascular endothelial dysfunction. In contrast, CBS-deficient humans with severe HHcy showed a higher risk of thrombosis, and patients with mild HHcy also had an increased risk of arterial or venous thromboembolic events. This difference in prothrombotic risk highlights the need for a representative animal model to study the relationship between HHcy and CVD in humans.

Met is a key player in protein turnover and is vital for the growth and preservation of lean body mass. However, consuming too much Met or not getting enough B vitamins (B-12, B-6, riboflavin, and folate) can lead to higher plasma levels of HCy, which might exacerbate the vulnerability of CVD, especially in populations already at risk. There is still much to learn about Met, including how its supplementation can be advantageous for health without raising the likelihood of CVD occurring in sports, prenatal care, or therapeutic interventions for sarcopenia [44].

To examine the relationship between hyperhomocysteinemia (HHcy) and the progression of atherosclerosis, Apolipoprotein-E-deficient (apoE^−/−^) mice—an established pre-clinical model for this human pathology—were assigned to either a hyperhomocysteinemic diet (HHD) deficient in methyl donors and B vitamins or a control diet (CD) containing sufficient levels of micronutrients. The HHD was characterized by a low amount of methyl donors and vitamins, including folate, choline, vitamin B6, and vitamin B12, combined with an excess of methionine (Met). Severe HHcy was confirmed in the HHD group, after which the aortic atheroma volume was measured through high-field magnetic resonance imaging (MRI). Additionally, plasma, aorta, and liver samples were examined to assess intermediates involved in homocysteine metabolism, and a targeted metabolomic analysis of plasma has been carried out [45].

## 4. The Relationship Between Supplements and Homocysteine Level

The plasma concentration of Hcy results from the intricate interplay between dietary habits and genetic predispositions such as mutations or polymorphisms in the *CBS* and *MTHFR* genes. Mutations in the latter can cause severe MTHFR deficiency, with enzymatic activity dropping below 20%, leading to the manifestation of HHcy, homocystinuria, and low plasma folate levels [46]. Dietary deficiencies of vitamin B12, vitamin B6, and folate are the predominant causes of HHcy because they are involved in the pathways of remethylation and transsulfuration.

Folate, a water-soluble form of vitamin B9, refers to a group of chemically related compounds crucial for periods of rapid cell growth and division, and DNA and RNA synthesis. While it is most commonly associated with supporting pregnancy and fertility, it is increasingly acknowledged for its positive effects on cardiovascular health, mental well-being, and cognitive function [47].

As other vitamins, folates cannot be produced in mammalian cells and are delivered from exogenous sources, namely foods and intestinal microbiota. In foods polyglutamylated FA, THF, 5-methyl-THF and 5,10-formyl-THF are ubiquitously present [47]. Animal liver and kidney, mushrooms, spinach, yeast, green leaves, and grasses are richest in folates. Foods enriched with FA and dietary supplements are the main sources of dietary folate.

FA remains inactive in the human body until it undergoes conversion in the liver, where it is modified into its biologically active form, known as 5-methyltetrahydrofolate (5-MTHF).

This compound acts as a methyl donor in an assortment of metabolic processes, such as the production of DNA precursor molecules, the synthesis of glycine from serine, and the conversion of Hcy into Met [48].

In this regard, several studies discussed the advantages and disadvantages of folate supplementation with FA versus 5-MTHF, from a different perspective [48].

Akoglu et al. conducted a double-blind, placebo-controlled study to evaluate the efficacy of L-5-MTHF (L-5-MTHF; 1 mg), FA (1 mg), and placebo in treating HHcy in liver transplant recipients. The study aimed to compare the relative responsiveness of these patients to L-5-MTHF and FA [49] and reported a significant reduction in total serum Hcy level exclusively in the L-5-MTHF group over the study period. No significant decrease in total serum Hcy level was observed in either the FA or placebo groups. The findings indicate that L-5-MTHF is significantly more potent than FA in lowering serum Hcy levels in liver transplant recipients, demonstrating its effectiveness in this context.

Lu et al. [50] investigated the combined impact of resveratrol and FA on blood pressure in spontaneously hypertensive rats with HHcy. Their study revealed that both compounds effectively reduced oxidative stress and enhanced the expression of endothelial nitric oxide synthase, even if resveratrol demonstrated stronger antihypertensive effects, likely due to its capacity to suppress angiotensin II expression.

Synthetic folic acid (FA), commonly found in fortification products and nutraceuticals, undergoes a two-step reduction process—first to dihydrofolate and subsequently to tetrahydrofolate (THF)—mediated by the enzyme dihydrofolate reductase (DHFR), in order to be integrated into the active cellular folate pool. When folic acid intake exceeds the enzymatic capacity of dihydrofolate reductase (DHFR), unmetabolized folic acid (UMFA) begins to accumulate in the plasma. The presence of circulating UMFA is, therefore, a hypothetical indicator that the body’s ability to convert FA into metabolically active folate has been exceeded [51].

The growing concern over folic acid (FA) and its correlation with UMFA syndrome has gained attention since UMFA is presently detected in the fetal umbilical cord and in the blood of babies [52].

The worry over FA and its correlation with UMFA syndrome has escalated. There is limited understanding of what constitutes excessive folate intake in humans and research models, as dose-response data and clear documentation of adverse effects at high folate levels are lacking. The recommended daily allowance (RDA) for FA varies by life stage, with 400 µg dietary folate equivalent (DFE) suggested for adult men and women, 500 µg for breastfeeding women, and 600 µg for pregnant women [53,54]. The tolerable upper intake level is set at 1000 µg per day for adults, but this was based on limited, low-quality data. Generally, folate intake exceeding the adult UL is considered excessive. Furthermore, the dosage of FA should be tailored based on patients’ clinical profiles, *MTHFR* mutation and anamnesis to ensure optimal outcomes.

In this context, Huang et al. demonstrated the efficacy of FA at a daily dosage of 0.8 to 1.2 mg in a cohort of 2697 hypertensive adults with elevated total Hcy (≥10 mmol/L) and no prior history of stroke or CVD [55].

Folate and vitamin B12 treatment improved insulin resistance and endothelial dysfunction, along with decreasing Hcy level, in patients with metabolic syndrome, suggesting that FA has several beneficial effects on CVD risk factors.

Vitamin B12 is a cofactor in the synthesis of Hcy from Met; therefore, deficiency of vitamin B12 can lead to HHcy. Studies have shown that deficiency of folate, vitamin B6, and vitamin B12 can cause dyslipidemia, vascular endothelial dysfunction, glucose intolerance, and insulin resistance through oxidative stress. Their deficiency leads to systemic inflammation and impaired nitric oxide synthesis, all of which are implicated in the pathophysiology of Metabolic Syndrome (MetS) [56]. Higher vitamin B12 level is inversely associated with MetS, whereas higher Hcy level is associated with MetS, anyway studies assessing the pathways underlying this association are required [56].

Vitamin B12, also known as cobalamin, is a cobalt-based nutrient produced by bacteria, crucial for mammals, who obtain it through their diet. Its absorption and distribution involve intricate biological mechanisms, including specific proteins, receptors, and transporters. If any step in this process is disrupted, it can result in vitamin B12 deficiency, potentially causing blood-related and neurological disorders [57].

Meat, eggs, and dairy products are the main sources of vitamin B12 intake; each person’s daily average intake is estimated to be 2.4 µg and people in a number of European nations do not consume enough FA and vitamin B12 to meet recommended levels [58].

In addition to inadequate consumption, other variables, including drug-nutrient interactions, malabsorption, certain medical conditions, or higher requirements, can result in vitamin deficiencies [59]. B vitamin deficiency is more common in older adults, with a prevalence of vitamin B12 deficiency estimated to be between 10 and 38% [60], and among vegans, as they consume less meat and dairy e [61].

To examine the effect of vitamin B12 supplementation on Hcy level, Sohouli et al. conducted a meta-analysis using 21 RCTs (N = 1625 individuals) [62]. The authors confirmed the effectiveness of supplementation, particularly when intervention periods ≥ 12 weeks and doses >500 µg/d, and indicated that hydroxocobalamin was more beneficial than other forms of vitamin B12.

In 1998, the Homocysteine Lowering Trialists’ Collaboration conducted a meta-analysis to evaluate the effect of folate supplementation’s impact on serum Hcy concentrations, with or without vitamins B6 and B12. They analyzed 12 trials with 1114 subjects, who had a mean age of 52 years and were treated for an average of 6 weeks. Folate supplementation lowered serum Hcy level by 25%. The addition of vitamin B12 produced a further 7% reduction, but vitamin B6 showed no significant additional effect [63].

The impact of several dietary supplements on lowering Hcy levels was recently assessed in another study [64]. The authors used a random-effects model to pool the data; direct and indirect evidence were integrated to do a network meta-analysis. This analysis comprised 16 studies in total. The scientists showed that lowering the Hcy level is more successful when FA is taken in combination with other vitamins, especially when the amount is near 800 μg. The optimal mixture is thought to be 1 mg of FA, 7.2 mg of B6, and 20 μg of B12.

All these data confirmed that vitamin B12 supplementation, especially if combined with other supplements, is effective in counteracting HHcy.

Omega-3 PUFAs and B vitamins are linked to the development of metabolic and degenerative disorders, such as CVD and cognitive impairment. Over the last two decades, extensive research has emphasized their interrelationship, indicating that PUFAs may influence the regulation of enzyme expression within the Hcy metabolic pathway. Additionally, the methylation process is regarded as essential for the proper metabolism and distribution of PUFAs throughout the body [65].

Some evidence indicates that omega-3 PUFA supplementation may influence the activity of enzymes involved in Hcy metabolism and contribute to the management of HHcy. However, these findings remain inconsistent in humans, necessitating further research to validate the results [66].

Although the limited data on this topic precludes definitive conclusions, it is hypothesized that a synergistic interaction between PUFAs and B vitamins could play a pivotal role in regulating key metabolic pathways. This interaction might account for the enhanced reduction in Hcy level observed when omega-3 PUFAs and B vitamins are co-supplemented. However, further research is needed to confirm the role of PUFAs in the management of HHcy.

Some therapeutic approaches are available to lower Hcy levels, including vitamin B supplementation, betaine administration, and multivitamin therapy [67,68]. Studies primarily focus on the cardiovascular benefits of reducing Hcy. According to the American Heart Association’s guidelines, FA supplementation at doses ranging from 0.2 to 15 mg per day effectively reduces Hcy levels, with a recommended daily dose of 0.8 mg for maximum reduction [69].

A network meta-analysis revealed that combining FA with vitamins B6 and B12 offers greater stroke prevention compared to FA alone or its combination with B12. Elevated Hcy levels are associated with an increased risk of coronary heart disease (7% for each 1 µmol/L increase), cerebrovascular diseases (59% for each 5 µmol/L increase), and mortality (33.6% for each 5 µmol/L increase) [70,71,72,73].

Folic acid supplementation has been shown to effectively reduce mild elevations in plasma homocysteine levels, improve endothelium-dependent vasoreactivity, and contribute to cardiovascular disease prevention. However, recent research highlights potential toxicities and adverse health effects associated with excessive folic acid intake, often arising from fortification or supplementation. Elevated folate levels may negatively impact health by influencing one-carbon metabolism, which plays a critical role in DNA replication and cell division, potentially contributing to cancer development [74].

In this regard, Vezzoli et al. (2020) investigate the effect of Oxoproline (Oxo), which, through the production of glutamic acid, provides an increase in intracellular folic acid trapping in moderate hyperhomocysteinemic (18.6 ± 2.4 μmol·L^−1^) patients suggesting that the use of oxoproline could be and intriguing alternative to traditional treatments for HHcy, when administered at doses below three grams per day, as approved by the European Food Safety Authority [75]. This effect is likely due to its role in promoting the intracellular synthesis of glutamic acid and polyglutamates. Furthermore, the absence of folates in the supplement eliminates the risk of an excess of unmetabolized folic acid, which could pose a long-term health concern. Nevertheless, additional research is necessary to further understand and validate the findings of this study.

Although various recommendations exist regarding supplements and their dosages to mitigate HHcy, further clarification is needed to identify effective measures to lower Hcy levels for disease prevention in healthy adults.

In a recent systematic review and network meta-analysis, Liu et al. (2025) evaluated the effectiveness of various nutritional supplements in lowering Hcy levels among healthy adults [64]. The analysis of 16 studies revealed that supplement combinations were more powerful than individual supplements, with the combination of 1 mg of folic acid, 7.2 mg of vitamin B6, and 20 μg of vitamin B12 being identified as the most effective in reducing Hcy levels.

Among the doses of folic acid, 800 μg of folic acid proved to be the most optimal strategy. Nonetheless, careful consideration is warranted due to the limited data available on the application of this specific combination in healthy adult populations.

## 5. Gut Microbiota and Homocysteine

Currently, the literature still lacks a solid connection between gut microbiota and Hcy. In this regard, the main findings indicate that the gut microbiota impacts Hcy metabolism by influencing the production of folate biosynthesis and the availability of methyl groups [76,77,78]. Furthermore, some of the treatments proposed to counteract HHcy also seem to act by restoring a healthier bacterial community. Three general ways exist for dysbiosis to contribute to the onset of chronic disease: (1) gain of function dysbiosis, where pathogens and their functions are acquired or opportunistically overgrown; (2) loss of function dysbiosis, where health-protective bacteria and their functions are lost or suppressed; and (3) a combination of loss and gain of function dysbiosis [79]. Several studies have documented elevated Hcy levels in animal models of dysbiosis and human patients [80,81].

In their study, Rizowy et al. [82] examined the gut microbiota profile of individuals with homocystinuria, which is typified by elevated plasma levels of Met and total Hcy and compared it with that of healthy individuals. Alpha and beta diversity did not differ between the groups; however, patients with homocystinuria were more likely to belong to the *Eubacterium coprostanoligenes* group and less likely to belong to the *Alistipes*, Family XIII UCG-001, and *Parabacteroidetes* genera. It is unclear if these variations in microbial ecology are due to dietary, genetic, or therapeutic factors.

In patients with Parkinson’s disease, Rosario et al. recently discovered a significant correlation between plasma Hcy levels and the bacteria *Akkermansia muciniphila*, *Eubacterium* sp., *Subdoligranulum* sp., and *Clostridiales* Family XIII [78].

Studies on piglets demonstrated that even if the liver is the principal site of Met metabolism, also the gastrointestinal tissue metabolizes dietary Met, contributing to Hcy production [9]. A recent study by Li et al. [77] confirmed the important role of the gut microbiota in Hcy metabolism. The authors demonstrated that mice subjected to a diet rich in Met showed an increase in Hcy that was counteracted through the administration of antibiotics without altering the gene expression and activity of hepatic CBS and BHMT. The high Met diet significantly modified the gut microbiota. At a family level, *Lachnospiraceae* and *Rikenellaceae* decreased, while *Prevotellaceae* increased; furthermore, the abundance of *Erysipelotrichales* (especially *Faecalibaculum* and *Dubosiella)*, which might produce and secrete Hcy, inducing host HHcy, was higher in the HM fed mice, while *Rikenellaceae* (especially *Alistipes)* were lower. A high Met diet raised the relative abundances of *Dubosiella newyorkensis* and *Faecalibaculum rodentium* at the species level. Interestingly, mice given a high-fat diet had higher levels of *Faecalibaculum* and *Dubosiella* [83,84]. *Faecalibaculum rodentium* plays a crucial function in phospholipid production, as evidenced by a recent study that found a favorable correlation [85]. The de novo Met biosynthesis route, which is lacking from mammalian hosts but preserved in intestinal bacteria [86], involves the transfer of cysteine to homoserine, which then cleaves to generate homocysteine. The enzymes CGS and CBL catalyze these reactions [87]. In their investigation, Li et al. [77] discovered a positive correlation between the bacterial gene abundance of CBL and the abundance of *Faecalibaculum* and a positive correlation between the bacterial gene abundance of CGS and the abundance of *Dubosiella*.

Recent studies have highlighted that gut microbiota can influence Hcy levels through the production of metabolites and the modulation of host metabolic pathways. Additionally, alterations in the gut microbiota composition can affect the absorption and metabolism of B vitamins, consequently impacting Hcy levels [88]. In any case, the mechanisms by which gut microbiota can influence Hcy levels in different pathologies remain to be elucidated.

### 5.1. The Relationship Between Gut Microbiota, Diet and B Vitamins

The composition of the gut microbiota is influenced by diet and can vary significantly based on the types of foods consumed. For example, a fiber-rich diet promotes the growth of beneficial bacteria such as *Bacteroidetes*, while a diet high in fats and sugars can favor the growth of pathogenic bacteria [89].

Studies have demonstrated a strong correlation between the human microbiome, vitamin B6, vitamin B12, and folate levels. These vitamins are produced by the gut bacteria in addition to being derived from the diet.

The majority of *Bifidobacteria* strains, with the exception of *B. gallicum* and *biavatii*, possess the genes necessary for folate synthesis [90], while the majority of lactic acid bacteria are unable to synthesize folate, except for *Lactobacillus plantarum* [91]. Certain medications used to treat type 2 diabetes mellitus and insulin resistance, may also have an impact on microbial folate synthesis; for example, metformin decreases folate synthesis by increasing *Coenorhabditis elegans*, which in turn lowers blood folate levels [92].

*Bifidobacterium adolescentis* and *pseudocatenulatum* supplementation raises fecal folate levels, according to Strozzi et al. [93]. Furthermore, LeBlanc et al. reported that *Bifidobacteria* and lactic acid bacteria, which are present in fermentable dairy products, can synthesize vitamins de novo, especially group B vitamins like vitamin B12 and FA [94]. The impact of a 12-week multispecies probiotic supplementation (containing *Bifidobacterium bifidum* W23, *lactis* W51, *lactis* W52, *Lactobacillus acidophilus* W37, *brevis* W63, *casei* W56, *salivarius* W24 and *Lactococcus lactis* W19 *lactis* W58) on oxidative stress, inflammation, and lipid profiles in obese patients was assessed by Majewska et al. [95]. Along with improvements in lipidic, inflammatory, and antioxidant status, a notable drop in Hcy level was observed.

Dysbiosis, a disorder in which the microbial composition of the gut is disturbed, can result from an imbalance or shortage in these vitamins; thus, sustaining sufficient amounts of folate and vitamins B6 and B12 are necessary for a healthy microbiome, which in turn promotes general health.

A crucial component of folate absorption, which happens through both folate receptors and particular receptors, is the gut microbiota [91].

It should be considered that malabsorption is common in patients with inflammatory bowel disease (IBD), and they tend to avoid foods that are the primary sources of folate, such as fresh fruits and vegetables. Additionally, people who use sulfasalazine may develop an FA deficit. Consequently, FA supplementation can be beneficial for IBD patients, who are at a higher risk of FA insufficiency [96].

The gut microbiome of Parkinson’s disease patients was examined by Rosario et al. [78], and personalized community-level metabolic modeling indicates the microbial contribution to HHcy and folate insufficiency seen in patients.

The composition of the gut microbiota may be impacted by supplements intended to treat HHcy. For example, Gurwara et al. [97] examined the connection between the dietary intake of B vitamins and methyl donors (Met, betaine, and choline) and the community composition and structure of the gut microbiota associated with the colonic mucosa. They discovered that alterations in the microbial profile were associated with B vitamins: a higher dietary intake of vitamin B12, pyridoxine, and folate was associated with an increase in evenness and richness. Furthermore, a larger intake of pyridoxine, vitamin B12, and folate was associated with a higher abundance of *Verrucomicrobia* and *Alistipes*.

Zinno et al. [98] investigated the effects of supplementation with dairy matrices enriched in organic folates on serum Hcy amount and fecal microbiota. The supplementation restored Hcy levels in FD mice and revealed differences in hepatic SAM levels. Furthermore, the fecal microbiota of mice receiving folate-enriched fermented milk or other types of milk exhibited greater bacterial species diversity compared to a control diet containing synthetic FA. Specific and significant changes in fecal microbiota composition were observed, while HHcy was not linked to notable alterations. The findings suggest that food matrices enriched with natural folates could be beneficial in managing HHcy [98].

### 5.2. How Gut Microbiota Can Influence Homocysteine Levels?

**Figure 2:**
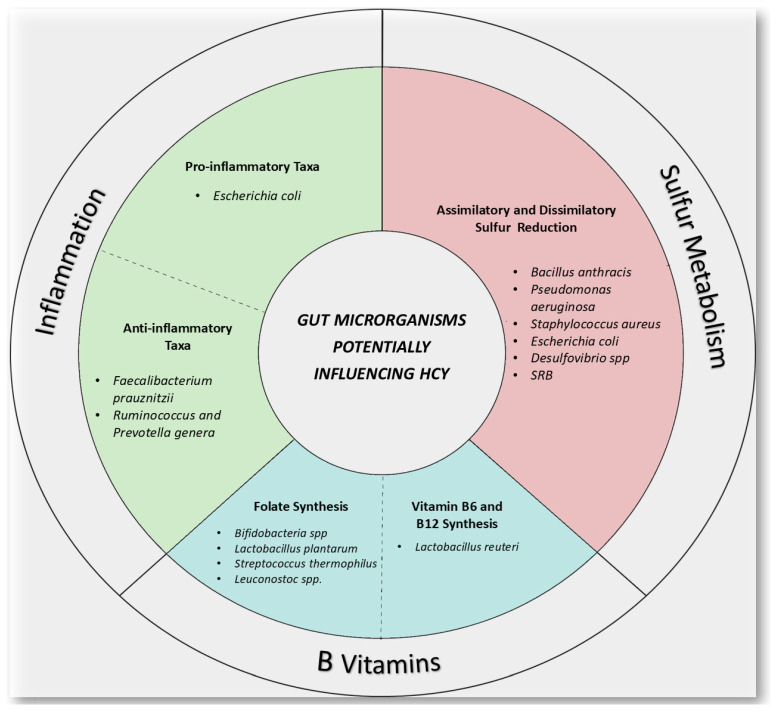
Schematic diagram of the mechanisms of gut microorganisms likely influencing homocysteine metabolism. The microbial species mentioned represent only a fraction of gut microorganisms involved in processes such as inflammation, sulfur reduction, and the production of B vitamins, which could impact homocysteine metabolism.

Despite the growing body of research on gut microbiota in recent years, evidence regarding the impact of intestinal bacteria on HHcy remains limited. As reported in the study by Li et al. [77], a positive correlation was observed between the abundance of *Faecalibaculum* and *Dubosiella* and plasma Hcy concentrations. Furthermore, the study revealed an increased presence of two KEGG (Kyoto Encyclopedia of Genes and Genomes) orthologies associated with Hcy biosynthesis within the gut microbiota of mice fed an HHcy-inducing diet. Some studies have identified an association between certain gut bacteria and Hcy levels, as previously reported, although the molecular mechanisms underlying these observations remain poorly understood. Nevertheless, potential mechanisms by which gut bacteria may contribute to the prevention or promotion of HHcy can be proposed. These mechanisms include vitamin production, sulfur metabolism, and modulation of inflammation. The diagram in Figure 2, far from being exhaustive, reports several bacterial taxa and their metabolic activity potentially affecting Hcy levels (Figure 2). As previously reported, most strains of *Bifidobacteria* [90,91,93] and *Lactobacillus plantarum* [94] exhibit the capacity to synthesize folate. Additionally, *Bifidobacteria* and lactic acid bacteria, commonly found in fermentable dairy products, have been shown to produce de novo vitamin B12 and folic acid [94,99,100].

The catabolism of sulfur-containing amino acids determines the formation of two crucial compounds: hydrogen sulfide (H_2_S) and Hcy. In addition to being essential for the transsulfuration pathway conversion of Hcy to cysteine, CBS and CGL are also in charge of the desulfuration processes that produce H_2_S. At physiological levels, H_2_S functions as a gaseous mediator with a variety of consequences, including the potential to modulate the metabolism of homocysteine [101]. Pathological diseases such as oxidative stress, inflammation, cardiovascular and brain dysfunction, fatty liver disease, and ischemia-reperfusion injury have all been linked to metabolic imbalances of Hcy and H_2_S [102]. Assimilatory sulfate reduction is common among microbes, allowing them to convert sulfate to sulfur for growth. However, only certain groups are capable of dissimilatory sulfate reduction. Sulfate-Reducing-Bacteria (SRB), such as *Desulfovibrio* species, are unique within intestinal ecosystems for their ability to use inorganic sulfate as an energy source [103]. These bacteria utilize sulfate as an electron acceptor, producing H_2_S. *Clostridium*, *Streptococcus*, and *Staphylococcus aureus* are examples of bacteria that break down cysteine via enzymes such as cysteine desulfhydrase, which releases H_2_S [104,105].

Wolf et al. revealed that genes involved in microbial sulfur metabolism are more abundant in the human gut than previously thought, regardless of health status. This discovery expands the known diversity of pathways and bacteria linked to sulfur metabolism in the gut [106].

Inflammation has been closely linked to HHcy. For instance, studies have demonstrated that elevated levels of interleukin-1 receptor antagonist (IL-1ra) and interleukin-6 (IL-6) exhibit significant associations with HHcy among elderly populations, thereby indicating a pro-inflammatory state correlated with heightened Hcy concentrations [107]. Furthermore, HHcy has been identified as a trigger for the activation of NLRP3 inflammasomes within macrophages, which subsequently leads to an increased production of pro-inflammatory cytokines such as interleukin-1β (IL-1β) and interleukin-18 (IL-18) [108].

A systematic review evaluated the interplay between gut microbiota composition and markers of low-grade inflammation in humans [109]. The findings revealed that reduced gut microbial diversity is associated with elevated white blood cell counts and higher levels of high-sensitivity C-reactive protein (hsCRP). Notably, certain bacterial genera, including *Bifidobacterium*, *Faecalibacterium*, *Ruminococcus*, and *Prevotella*, demonstrated inverse correlations with various markers of low-grade inflammation, such as hsCRP and IL-6 [110,111]. In contrast, *Escherichia coli* has been identified as having pro-inflammatory properties [112].

Recently a review reported multiple roles of *A. muciniphila*, on intestine inflammation, cardiovascular risk and obesity [113].

It should be noted that studies regarding the effect of different microbial taxa on inflammation reported in this section lead to the hypothesis of an effect also on Hcy levels, but studies investigating the direct effect of microbial bacteria on Hcy metabolism are few in number.

Even if the potential for gut microbiota modulation as a co-adjuvant in HHcy therapies is established, further human studies are needed in order to understand the real extent of the single strain effect and the underlying mechanisms.

Among the potential microbial mechanisms involved in Hcy synthesis and degradation, the involvement of the riboswitches-widespread conserved RNAs regulating metabolite levels in microbial cells through direct, noncovalent binding of their cognate metabolite-in the control of Hcy level in human gut microbiome cannot be excluded. Since most riboswitch-associated metabolites can be found in both the serum and fecal metabolome, the riboswitches might directly influence the host abundance of a wide range of molecules [114]. Riboswitches have been reported to regulate several cellular processes, including sulfur metabolism [115,116,117,118,119,120,121,122]. An increasing amount of evidence focuses on the riboswitches that sense SAM and SAH [122,123,124,125]. Further investigations are needed to elucidate the molecular mechanisms underlying the contribution of the gut microbiome to host Hcy metabolism.

## 6. Conclusions and Future Perspectives

Numerous conditions, such as cardiovascular illnesses, multiple sclerosis, diabetes, Alzheimer’s and Parkinson’s diseases, osteoporosis, and cancer, have been linked to HHcy [126]. HHcy is a risk factor also for CVD, as well as various neurological and psychiatric disorders.

Recent studies have identified a link between serum Hcy levels and gut microbiota in a range of neurological conditions, including schizophrenia, ischemic stroke, Parkinson’s disease and patients with major depressive disorder (MDD) [78,127,128,129]. The main clinical implications in this regard are summarized in Table 1.

Understanding the gut–brain relationship is crucial for cognitive function and decline across different conditions [132].

Considering the profound societal and economic burden of cardiovascular and cognitive impairments, developing innovative and efficacious treatments to counteract HHcy could be promising: the identification of gut bacteria signatures and their correlation with Hcy levels may open new avenues for microbiota-targeted therapies.

A balanced microbiota promotes metabolic health, modulates host immune function, reduces inflammation, and aids in maintaining gut mucosal integrity. Additionally, the gut microbiota plays a critical role in the de novo synthesis of essential vitamins including vitamin B12 and FA, and in sulfur metabolism, which significantly influences Hcy levels. Despite growing evidence in recent years of the complex interrelationship between Hcyand the gut microbiota, as well as the documented alterations of bacterial changes in people with HHcy and associated disorders, the true extent of the gut microbiota influence “in vivo” and the biological mechanisms underlying the effects remain unclear.

Numerous factors, including host genetics, nutrition, health state, aging, and the use of antibiotics, affect the gut microbiota composition, which has high inter-individual variability. Over the course of the human organism’s existence, the microbial community adjusts to these stimuli, adapting accordingly. The complexity of microbial species and influencing factors may account for the discrepancies observed in the literature, emphasizing the value of extensive longitudinal human studies and pointing to the necessity of a “multi-omic” approach that integrates diverse datasets from multiple sources rather than focusing solely on one factor.

The gut microbiota not only influences the health and metabolism of the host but also the response to any treatments, both at the level of supplementation and pharmacological therapies, potentially explaining therapeutic non-responsiveness or adverse effects in some patients. In the context of precision medicine, identifying microbial and metabolic features of the patients will help in designing tailored preventive and therapeutic strategies.

Restoring a favorable microbiota is a key goal in conjunction with bacterial characterization for individualized interventions. For this reason, several methods have been developed to manipulate gut bacteria to prevent or reverse unhealthy states; these include fecal microbiota transplantation, supplementation with specific strains probiotics, prebiotics, and supplements, and personalized nutrition. However, the composition of the gut microbiota in response to therapies and diet varies greatly between individuals, making this a challenging task. Ensuring the stability of the restored microbiota requires the application of well-designed methodologies, because, although the microbiota evolves and reacts to stimuli, it tends to return to its initial state when these stimuli are absent. Given the complexity of variables like individual characteristics, diet, supplementation, interventions and medications that can affect human biology, scientific researchers face significant challenges in advancing understanding of the relationship between gut microbiota and Hcy metabolism and translating this knowledge into clinical application. Nonetheless, these efforts could represent an invaluable opportunity to prevent and treat HHcy and related diseases.

## Figures and Tables

**Table 1:** Main clinical implications on the correlation between gut microbiota and Hcy levels in different diseases.

Clinical Implications	Gut Microbiota Taxa	Clinical Evidence	Reference
Major depressive disorder (MDD) vs. Healthy Controls	Negative correlation of Hcy with *Alistipes*, *Ruminococcaceae*, *Tenericutes*, and *Porphyromonas* and positive correlation with *Megasphaera* Negative correlation of Hcy with *Coprococcus* and negative relationship to *Pelomonas*, *Lachnospiraceae*, and *Tenericutes* Positive correlation of Hcy with *Lachnospiraceae* and *Escherichia*	In patients with MDD, elevated blood Hcy levels have a negative correlation with cognitive performance. This association could be linked to modifications in the gut microbiomal community structure.	[130]
Post-stroke cognitive impairment (PSCI) vs. post-stroke non-cognitive impairment (PSNCI)	PSCI group showed high *Proteobacteria* amount when compared to PSNCI group In aged-matched PSCI group, *Firmicutes*, including *Clostridia*, *Clostridiales*, *Lachnospiraceae*, and *Lachnospiraceae_other*, significantly decreased when compared to the PSNCI group	Gut microbiota was closely associated with Montral Cognitive Assessment (MoCA) scores and the risk factors for PSCI, including Hcy	[128]
Coronary artery disease (CAD) with and without HHcy vs. healthy controls.	*Clostridium cluster IV* and *Butyricimonas* were more abundant in CAD patients with HHcy with respect to CAD patients without HHcy	Patients with HHcy had a higher atherosclerotic burden linked to specific metabolites (BHMT, S-methyltransferase and trimethylamine N-oxide related). Patients with CVD who have HHcy, exhibit a higher atherosclerotic burden, poor Hcy metabolism, and an increased risk of cardiovascular disease, due to certain metabolites and an imbalance in gut microbiota	[127]
Early-stage levodopa (L-DOPA)-naive PD patients vs. matched PD and Healthy controls	Microbiome of patients with PD was clustered into *Bacteroides* and *Firmicutes* enterotypes and was associated to richness. There was no *Prevotella* enterotype in PD samples in contrast to the controls. PD patients had lower levels of *Paraprevetella clara*, *Prevotella* sp., and *R. intestinalis* than controls. In PD patients, *A. muciniphila*, *Subdoligranulum* sp., *Eubacterium* sp., and *Clostridiales family XIII* were increased, where have been identified as the main producers of Hcy, compared to controls	Personalized community-level metabolic modeling shows the microbial contribution to folate deficiency and HHcy	[78]
Schizophrenia (SZ) patients vs. healthy controls	Hcy levels were positively associated with *Eubacterium*, *Lactobacillus*, *Corynebacterium*, *Mogibacterium*, and *Bulleidia* in SZ patients	Cognitive function and gut microbial species are associated with blood Hcy levels	[131]

## Abbreviations

The following abbreviations are used in this manuscript:

Hcy	Homocysteine
Met	L-Methionine
PLP	pyridoxal-5′-phosphate
SAM	S-adenosylmethionine
MTHFR	methylenetetrahydrofolate reductase
CBS	cystathionine β-synthase
HHcy	hyperhomocysteinemia
CVDs	cardiovascular diseases
FA	folic acid
MS	methionine synthase
EC	endothelial cells
GIT	gastrointestinal tissue
MAT	ATP-L-Methionine S-Adenosyltranferase
SAH	S-Adenosyl homocysteine
AHCY	S-adenosyl homocysteine hydrolase
THF	tetrahydrofolate
5-methyl-THF	5-N-methyl tetrahydrofolate
BHMT	betaine-homocysteine methyltransferase
CGL	cystathione γ-lyase
SAT	O-acetyl transferase
OASS	O-acetylserine sulfhydrylase
OCBSs	O-acetylserine dependent CBSs
ACS	acute coronary syndrome
MD	Mediterranean Diet
MUFAs	monounsaturated fatty acids (MUFAs)
PUFAs	polyunsaturated fatty acids
GSH	glutathione
GSSG	oxidized glutathione
LBM	Lean body mass
apoE	Apolipoprotein-E
HHD	hyperhomocysteinemic diet
CD	control diet
5-MTHF	5-methyltetrahydrofolate
DHFR	dihydrofolate reductase
UMFA	unmetabolized FA
RDA	recommended daily allowance
DFE	dietary folate equivalent
MetS	Metabolic Syndrome
IBD	inflammatory bowel disease
MDD	major depressive disorder
